# Intake of Boron, Cadmium, and Molybdenum enhances rat thyroid cell transformation

**DOI:** 10.1186/s13046-017-0543-z

**Published:** 2017-06-02

**Authors:** Emilia Luca, Laura Fici, Anna Ronchi, Ferdinando Marandino, Esther Diana Rossi, Maria Emiliana Caristo, Pasqualino Malandrino, Marco Russo, Alfredo Pontecorvi, Riccardo Vigneri, Fabiola Moretti

**Affiliations:** 1 Institute of Pathology and Postgraduate School of Endocrinology, Catholic University of Roma, Rome, Italy; 2 Institute of Cell Biology and Neurobiology, National Research Council of Italy (CNR), Via del Fosso di Fiorano, 64, 00143 Rome, Italy; 3 National Center of Tossicology, IRCCS Foundation Salvatore Maugeri, Pavia, Italy; 4 Department of Surgical Pathology, “Regina Elena” National Cancer Institute, Rome, Italy; 5 Endocrinology, Department of Clinical and Experimental Medicine, University of Catania, Garibaldi-Nesima Medical Center, Via Palermo, 636, 95122 Catania, Italy; 6 Institute of Biostructure and Bioimaging, (CNR), Catania, Italy; 7 “Regina Elena” National Cancer Institute, Rome, Italy

**Keywords:** Thyroid, Thyroid nodules, Thyroid cancer, Heavy metals, Metalloids, Boron, Cadmium, Molybdenum, Volcanic area

## Abstract

**Background:**

Epidemiologic data in volcanic areas suggest that environmental factors might be involved in the increase of thyroid cancer (TC) incidence. Recent reports indicate that several heavy metals and metalloids are increased in volcanic areas. This study aims to evaluate the combined effect of three of these elements Boron (B), Cadmium (Cd), and Molybdenum (Mo) - all increased in the volcanic area of Mt. Etna, in Italy - on thyroid tumorigenesis in the rat.

**Methods:**

Female Wistar rats prone to develop thyroid tumors by low-iodine diet and methimazole treatment received *ad libitum* drinking water supplemented with B, Cd, and Mo at concentrations in the range found in the urine samples of residents of the volcanic area. At 5 and 10 months animals were euthanized, and their thyroid analysed. Statistical analysis was performed with a 2-way unpaired *t*-test.

**Results:**

No toxic effect of the three elements on the growth of the animals was observed. A significant increase of histological features of transformation was observed in thyroid follicular cells of rats treated with B, Cd, and Mo compared with those of control group. These abnormalities were associated with decreased iodine content in the thyroid.

**Conclusions:**

This study provides the evidence that slightly increased environmental concentrations of B, Cd, and Mo can accelerate the appearance of transformation marks in the thyroid gland of hypothyroid rats.

## Background

Thyroid cancer (TC) incidence has continuously and sharply risen since the early 1990s, more than any other cancer type. At present, TC is the 5^th^ most frequent cancer in women, whereas it ranked 14^th^ twenty years ago [1–3]. The causes underlying this increase are currently disputed. Some authors have suggested that this increase is apparent because of a more widespread use of screening and diagnosis of small tumors with minimal clinical relevance that went undetected in the past. Although a contribution of over-diagnosis is likely, some evidence indicates that a real increase is also occurring [4, 5]; indeed, the occurrence of large (>4 cm) thyroid tumors is also increased, and TC mortality rate is recently growing in spite of earlier diagnosis and better treatment.

Because of the rapid change observed in TC incidence, it is reasonable that environmental rather than genetic factors are involved. At present, the best known environmental factors able to promote thyrocyte growth and malignant transformation are radiation and iodine deficiency [6]. Radiation is a well-known risk factor for many cancer types, and the thyroid is a radiosensitive organ, especially at a young age, as demonstrated by the Chernobyl accident [7]. Chronic iodine deficiency represents a well-known determinant of follicular cell hyperplasia with thyroid hypertrophy (goiter) observed in both animal models and humans [8, 9]. The mechanism underlying the effects of low-iodine intake seems to rely on the increase of TSH levels that stimulate thyroid cells proliferation. In addition to radiation and iodine deficiency, environmental disruptors can also affect thyroid function and proliferation. For instance nitrates, frequent contaminants of water in areas of intensive agricultural industry can behave as potential thyroid function disruptor and carcinogen [10].

Epidemiologic studies performed in volcanic areas in different regions of the world have shown a marked increase in TC incidence, suggesting that unknown environmental factors of volcanic origin might contribute to the development of thyroid carcinoma [11–14]. These studies have also been performed in the volcanic area of Mt. Etna in Sicily, the largest active volcano in Europe, which has a large aquifer providing drinking water and irrigation for approximately 700,000 inhabitants. In this area, thyroid cancer incidence is doubled in comparison to the rest of Sicily and the increase is related exclusively to the papillary histotype [11].

In the same area, the levels of some heavy metals and metalloids are raised in drinking water and lichens compared with those of the adjacent non-volcanic areas. Human bio-contamination is demonstrated by the increase of these elements in the urine specimens of the residents, although with values in the normal range in most of the cases [15]. Within this multi-elemental pollution, it is hard to identify which element(s) may have a detrimental effect on thyroid function and transformation.

In this work, using a well-established *in vivo* model of thyroid tumorigenesis, we have undertaken a pilot study to analyse the effect on the thyroid of a combination of three elements: Boron (B), Cadmium (Cd), and Molybdenum (Mo). These elements are increased both in the environment (water and lichens) and in the urine samples of residents of the Mt. Etna volcanic area [15].

We have investigated the effect of chronic exposure to slightly increased concentrations of these elements (in the same range found in the urine samples of volcanic area residents) and, to better mimic the volcanic area conditions, the effect of each metal was not investigated individually but in combination with the other compounds. This choice was based on the assumption that, since average metal levels in the volcanic area are within the MAC (Maximal Admissible Concentration), the most likely hypothesis is that their combination rather than a single element may affect thyroid tumorigenesis.

The choice of these metals among the others increased in the volcanic area, was based on the availability of experimental data that suggest their association with altered thyroid function. More specifically:

i) B is the element at the highest concentration in the urine samples of the volcanic area residents and is one of the few chemicals whose increase in urines exceeded the urine reference limits in some individuals (over 20%) of the volcanic area population [15]. B has been reported to affect thyroid hormone concentration in gilts [16] and to be increased in the hair of children with goiter [17];
ii) Mo levels are over ten times increased in drinking water of the volcanic area compared with that of adjacent areas, and its urine concentration in volcanic area residents is higher than normal limits in more than 20% cases [15]. Mo has been reported to interact with the thyroid hormone receptor [18] and to significantly correlate with urinary iodine levels [19];
iii) Cd levels are more than ten times increased in the volcanic area water [15]; Cd is a well-recognized carcinogen (group 1 carcinogen according to the IARC classification) [20] although at concentrations higher than those used in this study. Cd has also been reported to accumulate in the thyroid [21] and, after chronic exposure, to be associated with increased thyroid hormone levels [22] and pre-neoplastic thyroid abnormalities [20]. Moreover, levels of Cd are higher in thyroid tissue of patients with advanced thyroid cancer [23].

## Methods

### Experimental design

To evaluate the effects of B, Cd, and Mo on thyroid tumorigenesis, we used a rat model prone to develop this cancer because of the treatment with methimazole and low-iodine diet, two well-known goitrogenic factors [24–28]. Female rats were chosen because of the increased rate of thyroid tumors in women [29]. The three elements were supplemented in the drinking water at a concentration double that observed in the urine specimens of residents of the Mt. Etna volcanic area (Table 1).

**Table 1:** Elements, compounds, and concentration [C] used for animal treatment

Element (MW^a^)	[C] of element in urine samples	[C] of element in drinking water of Group B	Compound used in drinking water (MW)	[C] of compound in drinking water of Group B	LD50 compound in rat
B (10.81)	800 μg/L (74 μM)	1600 μg/L (148 μM)	Boric Acid (61.83)	9.14 mg/L	270–675 mg
Cd (112.41)	0.2 μg/L (1.8 nM)	0.4 μg/L (3.6 nM)	Cadmium Chloride (183.32)	0.66 μg/L	8.8–22 mg
Mo (95.96)	50 μg/L (0.52 μM)	100 μg/L (1.04 μM)	Ammonium heptamolybdate tetrahydrate (1235.86)	1.286 mg/L	33–83 mg

^a^ *MW* molecular weight

Twenty-eight female Wistar rats (9 weeks old, 200–230 g) obtained from the animal service of the Catholic University of Rome, were kept under standard housing conditions (temperature 21°–23 °C, relative humidity 45–65%, and 12 h:12 h light/dark cycle) with aseptic food and tap water *ad libitum*. All animals were housed in plastic cages containing two or three animals/cage. All rats but two were treated with drinking water containing 0.003% methimazole (MMI) and low-iodine diet (Remington’s Diet, Mucedola) to make them hypothyroid. The two untreated female rats were grown under the same housing conditions but without MMI and low-iodine diet and their thyroid was used as normal control for histological analysis. Experimental rats were randomly divided into two groups (11 rats/group A, 15 rats/group B). Control group A received drinking water containing 0.003% MMI (Sigma-Aldrich) and low-iodine diet. Rats in group B were given, in addition, two heavy metals (Cd, and Mo) and one metalloid (B) dissolved in drinking water at the concentration and under the chemical form reported in Table 1.

All animal experiments were approved by the ethical committee of the Catholic University of Rome, Italy (n. Q42), and were conducted in accordance with institutional guidelines, which are in compliance with national (D.L. No. 116, G.U., Suppl. 40, Feb. 18, 1992; Circular No. 8,G.U., July 1994) and international laws (EEC Council Directive 86/609, OJ L 358. 1, Dec 12, 1987; Guide for the Care and Use of Laboratory Animals, United States National Research Council, 1996) on the ethical use of animals.

### Sample collection

Clinical observation of animals was carried out daily, and body weight was measured every 2 weeks. After 1, 5 and 10 months from the beginning of treatment, three rats from both control and treated groups were housed in metabolic cages, and 24 h urine samples were collected and stored at -80 °C for iodine and metal analysis.

After 5 and 10 months, 5 and 6 rats from Group A, and 5 and 10 rats from Group B, respectively were anesthetized with Ketamine (75 mg/kg i.m.) and medetomidine (0.5 mg/kg i.m.) and killed by exsanguination. Thyroid portions were collected as follows: a) formalin-fixed-paraffin-embedded (FFPE) tissue sample for histopathological examination; b) tissue specimen stored at -80 °C immediately after harvesting for subsequent iodine and B, Cd and Mo analysis.

### Histopathological analysis

FFPE tissue samples were cut into 3 micron thick sections and stained with hematoxylin-eosin. Evaluation of thyroid morphological parameters was based on the analysis of architectural and cellular features. The following parameters were evaluated by two pathologists blinded to the animal treatment with elements. The criteria used are in keeping with the histological evaluation of the human thyroid and are based on previous reports on the morphology of rat thyroid [24–26]. The thyroid parameters evaluated were:

1. Follicular size (size less than 0.5 mm was classified as micro);
2. Cellular morphology: ranging from flat in normal thyroid, to cuboidal with hyperplastic or neoplastic features. Hyperplastic features are defined by the presence of nuclear clearing and/or granular inclusion; neoplastic features are defined by nuclear clearing with the presence of pseudo-inclusions;
3. Disomogeneity (defined as nodular hyperplasia for the presence of parenchyma with either pseudonodular or nodular pattern and with hyperplastic features characterised by oxyphilic cellular changes and some nuclear/cytoplasmic clearing and pseudo-inclusions);
4. Presence of oxyphilic cells, and/or granular cells;
5. Nuclear clearing;
6. Vascular congestion;
7. Papillary structure;
8. Presence of inflammation features

The diagnosis of papillary thyroid carcinoma was based on the presence of papillary structures with distinctive nuclear features (pleomorphic nuclei, grooves, nuclear pseudo-inclusion) and micro or medium-follicular structures with evidence of capsular invasion.

Pictures were analysed by optical microscope BX53F (Olympus Corp., Tokyo, Japan).

### Element analysis

Elements were analysed in urine and thyroid tissue samples. Urine specimens were thawed, thoroughly mixed, filtered and appropriately (3- to 9-fold) diluted with ultrapure acidified water (1% HNO_3_). Tissue samples underwent a pre-treatment in microwave to reduce interference caused by the matrix; the use of a microwave assures rapidity of execution and an accurate control of accidental pollution phenomena. Digestions were carried out in a microwave sample preparation system CEM (Corp., Matthews, NC, USA) Model MARS-Xpress equipped with a 1400 W magnetron that is adjustable with 1% increment and operating at a microwave frequency of 2455 MHz. Digestion vessels were cleaned with 10 mL HNO_3_ using the microwave cleaning program and were rinsed with deionized water supplied by a Milli-QTM Laboratory Water System (Millipore). The operating conditions for the Microwave Digestion were: Step 1, Power 240, hold time 2 min; Step2, Power 360, hold time 2 min; Step 3, Power 480, hold time 15 min. Tissue samples (approximately 50 mg) were added to the digestion vessels with 4 mL HNO_3_ (65% m/v) and 0.5 mL H_2_O_2_ (30% m/v). After complete digestion and cooling, the samples were filtered and diluted with deionized water. Measurements of trace elements were performed using an Inductively Coupled Plasma Mass Spectrometer (ELAN 6100 DRCII ICP-MS; Perkin-Elmer SCIEX Instruments, Concord, Ontario, Canada) equipped with a cyclonic spray chamber and concentric nebulizer, a quadrupole mass filter and a dynamic reaction cell (DRC). This instrument allows obtaining background values <1cps and detection limits in the order of ng/L. Moreover, the mass spectrometer allows working in a linear way in a wide dynamic range, by analysing the various analytes at different concentrations with comparable precision and accuracy. The dynamic reaction cell can be pressurized with the reactive gas (CH_4_, NH_3_) and provides online chemical modification of the ion beam for eliminating spectroscopic interferences. The specificity of interference rejection is obtained through the selection of the reaction gas and the operating conditions. In the vented “standard mode” no reaction gas is present in the cell, and the instrument shows the typical characteristics of a quadrupole-based ICP mass spectrometer. B, I and Mo, were determined in standard mode while Cd in enhanced mode (DRC). Instrumental conditions are summarized in Table 2. The validation process demonstrated linearity, sensitivity, precision and accuracy of the method.

**Table 2:** Instrument Operating Parameters for DRC-ICP-MS analysis

RF power	1250 W	
Plasma argon	15 L/min	
Nebulizer flow	1.0 L/min	
Auxiliary flow	1.3 L/min	
Sample flow rate	1 L/min	
Nebulizer	Mainhart	
Interface cones	Platinum	
Scan mode	Peak hopping	
Resolution	Normal	
Sweeps/readings	20	
Readings/replicates	3	
Number of replicates	5	
Sample time	1 min 39 s	
Sample read delay	50 s	
Autosampler wash delay	45 s	
Calibration mode	External calibration	
Calibration standard	0.1, 0.5, 1, 2, 5, 10, 20 μg/L	
Curve fit	Linear through zero	
DRC parameters	DRC Vented (standard mode)	DCR Pressurized (enhanced mode)
Cell q parameter	0.25	0.65
Cell a parameter	0	0
Cell gas	No gas	NH_3_ at 0.5 mL/min

Since the thyroid gland includes a liquid phase (colloid) in addition to the solid phase (cellular), with the possibility of variations of the ratio of the two phases after treatment, measured element values were normalized by both tissue weight and DNA content.

### Statistical analysis

Statistical analysis was carried out with the Analyse-it software, version 2.22, Microsoft Excel (Analyse-it Software, Ltd.) by 2-way unpaired *t*-test. A *p*-value <0.05 was considered statistically significant.

## Results

### Boron, Cadmium, and Molybdenum do not cause overt toxicity

**Fig. 1:**
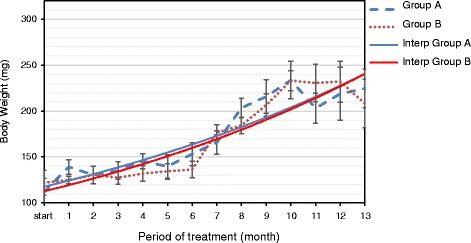
Metal supplementation does not alter animal growth. Weight curves of control group A (*dotted blue line*) and metal-treated group B (*dotted red line*). Continued lines represent the linear interpolation of experimental values from group A (*blue line*) and metal-treated group B (*red line*)

No significant difference in food and drinking water consumption was observed in the rats treated with B, Cd, and Mo compared with that of control group A. Consistently, similar body weight was observed in the two groups (Fig. 1), indicating that these elements, at the low-dose used, do not cause signs of evident toxicity in these animals. Accordingly, no clinical evidence of toxicity has been reported in individuals living in the Mt. Etna area.

**Fig. 2:**
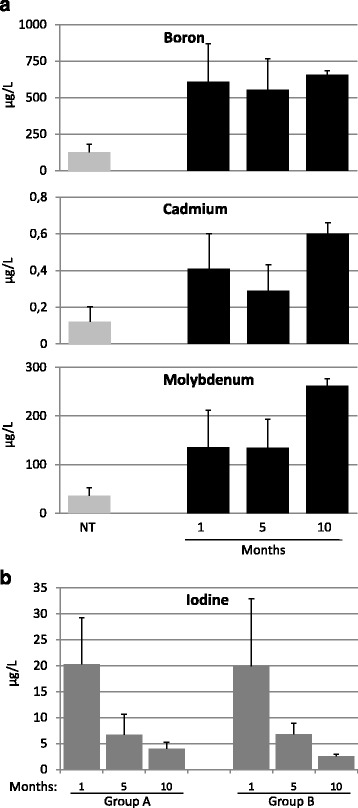
Supplemented metals accumulate in the urine samples of experimental rats. **a** Urine values of B, Cd, and Mo in the control group A (NT) and metal-treated group B after 1, 5 and 10 months of treatment. **b** Urine values of iodine in the control group A (NT) and metal-treated group B after 1, 5 and 10 months of treatment. Error bars represent Mean ± SD (*n* = 5)

To evaluate B, Cd, and Mo consumption by the animals, we measured the levels of these elements in rat urine samples at 1, 5 and 10 months after the beginning of the treatment. The levels of all three elements were significantly increased in the urine samples of the treated animals compared with those of the control group A (NT) confirming that these metals had been ingested and absorbed (Fig. 2a). Specifically, urinary Cd and Mo were at concentrations similar to those of drinking water (0.4 μg/L and 100 μg/L, respectively) and approximately two-fold higher than those found in the urine samples of the volcanic area residents (Table 1). In contrast, B concentration in the urines samples of treated rats, was slightly lower than that present in drinking water (1600 μg/L), suggesting a different retention/excretion rate of this metal in the rat. Analysis of iodine in the same urine samples confirmed that iodine content was progressively and similarly decreased in the two groups as a consequence of the low-iodine diet (Fig. 2b).

### Boron, Cadmium, and Molybdenum accelerate and worsen thyroid abnormalities induced by goitrogenic diet

To investigate the effect of metal supplementation on the thyroid, animals were sacrificed at two experimental time points, 5 and 10 months after the beginning of treatment, and their thyroid gland was analysed. Thyroids of the A and B groups were also compared with those of healthy untreated rats.

As a consequence of the hypothyroidism induced by the goitrogenic diet, the thyroid of both A and B groups was markedly enlarged compared with that of untreated rats in agreement with previous report [25, 27]. No difference was observed in the thyroid volume between groups A and B.

**Fig. 3:**
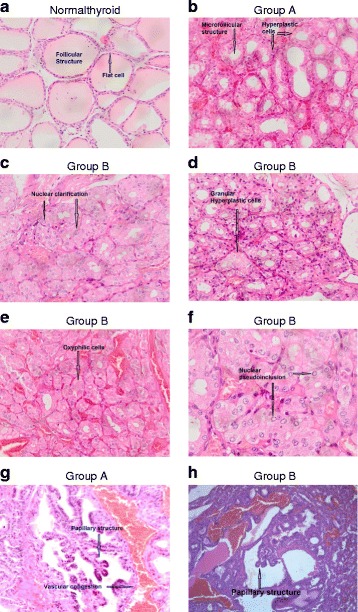
Representative picture of hematoxyline-eosin staining of thyroid specimens from a normal untreated rat **a**, a Group A rat treated with the goitrogenic diet **b** and **g**, and Group B rats treated with the goitrogenic diet and B, Cd and Mo **c-f** and **h**. Arrows indicate thyroid features as reported in Table 4

Histological analysis of the thyroid from healthy untreated animals showed a uniform architectural pattern, with homogenous follicular structures characterised by flat cells without vascular congestion. (Fig. 3a).

In comparison, thyroid from both A and B groups showed an irregular follicular pattern with microfollicoli (size less than 0.5 mm) and cuboidal cells (Fig. 3b). Also, a variable amount of vascularization was present (Fig. 3b-h, Table 3).

**Table 3:** Cumulative morphological features of the thyroid in group A and B

	Features	Groups^a^	
		A	B
5 Months			
	Follicles		
	- Disomogeneity (mild)	0%	40% (2/5)
	- Nuclear clearing	0%	40% (2/5)
	- Papillary structures	0%	0%
	Vascular congestion (mild)	100% (5/5)	100% (5/5)
10 Months			
	Follicles		
	- Disomogeneity (mild)	17% (1/6)	0%
	• (moderate)	83% (5/6)	50% (5/10)
	• (severe)	0%	50% (5/10)
	- Nuclear clearing (mild)	17% (1/6)	30% (3/10)
	• (moderate)	83% (5/6)	30% (3/10)
	• (severe)	0%	40% (4/10)
	- Papillary structures (mild)	17% (1/6)	10% (1/10)
	• (moderate)	0%	50% (5/10)
	• (severe)	0%	40% (4/10)
	Vascular congestion (mild)	0%	0%
	• (moderate)	83% (5/6)	50% (5/10)
	• (severe)	17% (1/6)	50% (5/10)

^a^number of affected/total animals

Mild = present in less than 10% of the examined area, Moderate = present in 10–30% of the examined area, Severe = present in more than 30% of the examined area

After 5 months, in 2 out of 5 rats of group B, additional disomogeneity of the follicles characterised by nodular hyperplasia with either pseudonodular or nodular pattern was observed. Cell morphology showed abnormal nuclear morphology with marks of clearing (Fig. 3c, Table 3). This last feature characterises both benign and malignant lesions and is a diagnostic marker of cell transformation when associated with diffuse well-defined papillary structures [30].

After 10 months of goitrogenic diet, the architectural pattern of the thyroid was further compromised with complete disorganization of the follicular structure, discrete amount of vascularization and increased number of hyperplastic, granular and oxyphilic cells in both groups (Fig. 3d-f, Table 3). In fact, the appearance of these cells is related to the hypothyroidism caused by MMI and low-iodine diet and characterises both benign and malignant thyroid lesions [31, 32]. In rats from group B, thyroid cells presented additional neoplastic features defined by the presence of granular and nuclear pseudo-inclusions.

The presence of papillary structures became evident in the thyroid of hypothyroid rats of both A and B groups, in agreement with previous reports (Fig. 3g-h, Table 3). However, the presence of these structures was markedly increased in the thyroid of group B (Table 3). In addition, in these animals the papillary structures were associated with the appearance of abnormal nuclear morphology and nuclear pseudo-inclusion, markers of cell transformation (Fig. 3f). No overt signs of inflammation were observed in the thyroid of the animals of both groups (Fig. 3).

To investigate the possible cause of this phenotype, we analysed iodine concentration in the thyroid of a subgroup of animals (depending on the available tissue). Of interest, in elements-treated animals thyroid iodine content was significantly decreased compared with that of control group when data were normalized to DNA content (*p* = 0.00997, Table 4), suggesting that iodine uptake and/or retention was negatively affected by metal supplementation.

**Table 4:** Concentrations of chemical elements in rat thyroid samples

Groups	DNA	I Iodine		B Boron		Mo Molybdenum		Cd Cadmium
		μg/g^a^	ug/DNA	μg/g	ug/DNA	μg/g	ug/DNA	μg/g
TA1	0.974	2.378	2.440	0.181	0.186	0.045	0.046	<0.01
TA2	0.440	1.039	2.361	0.342	0.778	0.036	0.083	<0.01
TA3	0.414	1.386	3.348	1.025	2.476	0.036	0.086	< 0.01
TA4	0.178	0.479	2.692	0.122	0.686	0.044	0.245	< 0.01
TA5	0.217	0.716	3.294	0.174	0.801	0.052	0.241	< 0.01
TA6	0.253	0.943	3.733	0.159	0.630	0.036	0.142	< 0.01
MEAN		1.109	2.978	0.334	0.926	0.041	0.140	
SD		0.742	0.468	0.376	0.870	0.007	0.095	
TB1	0.322	0.441	1.368	0.572	1.774	0.024	0.074	< 0.01
TB2	0.451	0.312	0.691	0.678	1.502	0.035	0.076	< 0.01
TB3	0.568	0.458	0.806	0.161	0.283	0.050	0.089	< 0.01
TB5	0.340	0.889	2.611	< 0.01		0.046	0.136	< 0.01
TB6	0.549	0.762	1.389	0.081	0.148	0.043	0.078	< 0.01
MEAN		0.572	1.373	0.373	0.915	0.040	0.091	
SD		0.652	0.761	0.296	0.830	0.010	0.0256	
P		0.134229	0.009976	0.854547	0.9994165	0.74384	0.222808	

^a^μg/g = μg of chemical/g of thyroid tissue

We, therefore, analysed whether the studied elements accumulate in the thyroid of metal-treated rats. However, the levels of B and Mo did not differ between Group A and B (Table 4), thus excluding a mechanism of competition/replacement. Cd levels were undetectable in both groups. Overall, these data indicate that the histopathological features observed in the thyroid of element-treated rats are not due to an increased concentration of the three elements and suggest that metal supplementation may interfere with iodine uptake.

## Discussion

Our study indicates that in Wistar female rats fed with a goitrogenic diet, additional administration of low levels of Boron, Cadmium, and Molybdenum causes an increase of thyroid abnormalities.

The goitrogenic diet, *per se*, has been widely reported to induce thyroid tumorigenesis [25]. Rats exposed to this diet, develop various features of cell transformation: nuclear aberrations, change of cell morphology and, most important, papillary structures. Whereas most of these features are present also in benign lesions, the presence of papillary structures in association with nuclear aberrations may be *bona fide* recognized as a marker of thyroid carcinoma. In control rats (group A), these features of malignant transformation were evident in only one animal after 10 months of goitrogenic diet. Conversely, in element-treated rats (group B), these alterations were more frequent, with the presence of papillary structures occurring in all animals of the group at the same time point. These data suggest that even a slight increase of Boron, Cadmium, and Molybdenum in the diet may accelerate and/or promote the process of cell transformation, thus acting as a tumor-promoting agent rather than a carcinogen. Indeed, in rats treated with low iodine diet, with or without a goitrogenic drug, thyroid tumors occur with high frequency after 18 months [26, 28] while when the low-dose elements were added to the diet, clear marks of follicular cell transformation were observed at 10 months.

The genotoxic effect of some metals is well recognized. Specifically, the overall carcinogenicity of Cd is well established [33]. Conversely, no similar evidence has been reported for B and Mo that may have rather a protective effect on genotoxicity through a variety of different mechanisms [34–38]. The few published data concern their activity on thyroid function and these functional data are often inconsistent and not comparable because of the different experimental models and different study design. To our knowledge, the effect of low concentration of these metals on thyroid carcinogenesis has never been reported.

In fact, a major novelty of our study is that the studied elements were supplemented in the μg or μM range and at concentrations within the non-toxic range while previous studies have used metals in the mg or mM range. Moreover, in most of the previous studies, the exposure to metals was usually short-lasting (days or weeks) whereas we examined low-dose chronic effects as occur in the residents of the volcanic area where thyroid cancer incidence is increased.

The molecular mechanism of the thyroid transforming activity of B, Cd, and Mo at low, non-toxic dose remains unclear. Our data show that these elements impair the iodine uptake from thyroid. However, the mechanism by which this occurs remains to be ascertained. One possibility is that the mixture of elements may enhance the specific detrimental effect of each chemical because of the synergistic consequences of disrupting multiple cellular mechanisms. Additionally, the reduced iodine content in the thyroid of element-treated rats could be due to decreased uptake and hormonogenesis due to the increased follicular cell transformation.

Our current understanding of heavy metals’ and metalloids’ effect on the thyroid is very limited because the toxicological profile of water-soluble chemicals and their tissue-specific tumorigenic potential is still insufficient. A quantitative, poorly-investigated mechanism, for instance, is the hormesis effect, a biphasic dose-response relationship already documented in vitro for different metals, including Cd [39, 40]. Hormesis is characterised by stimulation of biological effects at lower concentrations and inhibition at higher concentrations [39] and has important toxicological implications for the potential consequences of chronic exposure to very low levels of many toxicants [41]. The U-shaped immune function response characteristic of low and high doses of B supplementation in rats [42] could involve this mechanism. In this context, the reduced iodine content in the thyroid of elements-treated rats may play an additional role.

## Conclusions

In conclusion, chronic exposure of hypothyroid rats to B, Cd, and Mo at the levels found in drinking water of Mt. Etna volcanic area, causes an accelerated appearance of features of malignant transformation in thyroid cells. This study has to be considered an exploratory analysis suggesting a possible cause-effect relationship between thyroid cancer and the combination of the tested elements. Further studies will be necessary to ascertain the evolution of clinically evident thyroid tumors and the mechanism/s involved. Given the high prevalence of population exposure to metals in the industrialized world, this work warrants in-depth molecular studies on the activity of different metals in thyroid carcinogenesis.
